# Mobile Diagnostic Devices for Digital Transformation in Personalized Healthcare

**DOI:** 10.3390/diagnostics10121008

**Published:** 2020-11-25

**Authors:** Hatice Ceylan Koydemir, Aniruddha Ray

**Affiliations:** 1 Department of Electrical and Computer Engineering, University of California, 90095 Los Angeles, CA, USA; 2 Department of Physics and Astronomy, University of Toledo, 43606 Toledo, OH, USA

Mobile devices have increasingly become an essential part of the healthcare system worldwide. This is particularly evident during the current COVID-19 pandemic, as telemedicine is playing an important role in enabling remote interaction between physicians and patients, to support patient care and disease management, while maintaining social distancing. In addition to telemedicine, mobile devices have played a key part in evaluating and guiding the pandemic response, by facilitating contact tracing, as well as helping with mapping the transmission [1]. There has also been a significant effort towards the development of mobile diagnostic devices for point-of-care (POC) testing of COVID-19 in order to curb the pandemic. A few different POC devices for the rapid detection of the SARS-CoV-2 have been developed, including the Accula system (Mesa Biotech), Sofia 2 (Quidel), Talis One (Talis Biomedical) and Cue (Cue Health), which are expected to enable rapid testing and significantly increase the rate of screening. Thus, the use of POC diagnostic devices will play a key role during such pandemics, especially where vaccines are yet to be developed, by preventing the spread of the disease and reducing the mortality rates. Although promising, there are several challenges associated with the deployment of such devices. These include filing regulatory approvals in different countries worldwide, negotiating the terms of cost reimbursement from insurance companies, maintaining data privacy and the protection of data, among others. Despite the challenges, mobile devices offer several advantages that make them attractive to clinicians, patients, and even educators. One of the main advantages of mobile devices is their ability to provide healthcare access to patients in low-resource settings, such as rural areas or poor communities, at an affordable cost. Mobile devices can be used for on-site testing and data collection using their in-built cameras and sensors, as well as external lab-on-chip platforms, paper-based assays, and other formats of POC tests [2]. Advancement in processor technology has also facilitated complicated calculations and analysis, on the spot, using these mobile devices and simple user interfaces (e.g., mobile applications). Furthermore, the data acquired from on-site testing can also be uploaded to different servers worldwide, which would facilitate data analysis using computationally intense approaches, such as machine learning, deep learning, or even crowdsourcing. Another advantage of remote diagnostics and telemedicine is the reduction in patients’ exposure to nosocomial infections, which can be life-threatening (e.g., methicillin-resistant *Staphylococcus aureus* (MRSA)). The utilization of mobile devices such as smartphones, smartwatches, implantable sensors, and portable readers in healthcare has several benefits to public health strategies as well, especially for monitoring chronic medical conditions. For example, smartwatches can measure blood pressure in patients suffering from hypertension. Standalone medical devices, such as the glucometer, which is a standard tool for diabetes management, have become quite popular as well. Another important area is the detection of bacterial and viral pathogens, which was reviewed by Nath et al. [3] The pathogen detection devices are typically based on portable lab-on-chip platforms, such as microfluidics or plasmonics, and integrated with optical or electrochemical readers. This review focuses on the point-of-care optical readers, such as smartphone and holographic microscopes, with in vitro diagnostic assays that are easy to perform in resource-limited settings.

Dieng et al. [4] developed a mobile set-up, composed of a sample inactivation extraction station, sample preparation station, and detection station, for the detection of the causative agent of non-malaria febrile illnesses by screening for dengue virus (DENV), Zika virus (ZIKV), yellow fever virus (YFV), chikungunya virus (CHIKV), and rift valley fever virus (RVFV). They evaluated the performance of this mobile laboratory by screening the blood samples of 104 children in Senegal and confirmed their test results to gold standard molecular methods such as real-time polymerase chain reaction (RT-PCR) and enzyme-linked immunosorbent assay (ELISA). Another optical platform for virus detection, i.e., Zika virus, for which effective vaccines are not yet available, was demonstrated by Kabir et al. [5]. This mobile in vitro diagnostic platform is based on a smartphone-based video reader for a colorimetric assay. This device has a high throughput with a 9 min turnaround time, including sample preparation.

Smartphones can also be used in combination with smart wearable sensors and implants to collect patient data, which can then be used to predict different health problems, as well as monitor the dynamics of a particular illness [6]. Rodriguez-Riuz et al. [7] analyzed the night, day, and 24 h motor activity data of 55 patients, of which 23 were diagnosed with depressive episodes, to find the best dataset for the accurate classification of depressive episodes using machine learning and a smart wearable. They demonstrated that the night motor activity data were the best dataset for this classification with >99% specificity and sensitivity. Similarly, smartphones were also used to study neurological disorders and disabilities. Carmono-Perez et al. [8] studied the craniocervical range of motion in subjects between the ages of 4 and 14 with cerebral palsy, which is a neurological disorder that affects movement and motor skills. An inertial measurement unit (IMU) device and a cervical range of motion (CROM) device were used to perform the measurements of movement in different spatial planes. Both IMU and CROM had a high level of correlation between them for the assessment of craniocervical motion. In another example, Shichkina et al. [9] evaluated the effectiveness of the use of the mobile phone and neural networks for the determination of the status of patients with Parkinson’s disease. In this study, a smartphone was used to collect patient data, such as speech, hand tremors, tapping of fingers, speed, balance, and reaction time, which was used to train a deep recurrent neural network, that can help determine the condition of the patient. In a separate study, Rodriguez-Almagro [10] used a new virtual reality system, consisting of a mobile device and a headset, to perform subjective visual vertical (SVV) test, on subjects suffering from disabilities such as migraine and tension-type headache. However, this test did not yield any significant differences between the control group and the group with a disability, thereby highlighting the need to perform additional visual and somatosensory tests.

In conclusion, a number of interesting applications ranging from pathogen detection to neurological disorders and mental health problems were presented in this Special Issue, which highlights the capabilities of mobile diagnostic devices and their importance in personalized healthcare.
